# Brain lateralization in children with upper-limb reduction deficiency

**DOI:** 10.1186/s12984-020-00803-1

**Published:** 2021-02-03

**Authors:** Jorge M. Zuniga, James E. Pierce, Christopher Copeland, Claudia Cortes-Reyes, David Salazar, YingYing Wang, K. M. Arun, Theodore Huppert

**Affiliations:** 1 Department of Biomechanics, University of Nebraska at Omaha, Omaha, NE 68182 USA; 2 Department of Special Education and Communication Disorders (SECD), University of Nebraska-Lincoln, Lincoln, NE 68182 USA; 3 Department of Imaging Sciences and Interventional Radiology, Sree Chitra Tirunal Institute for Medical Science and Technology, Thiruvananthapuram, India; 4 Department of Radiology, University of Pittsburgh Medical Center, Pittsburgh, PA 16148 USA

**Keywords:** Upper-limb deficiency, Prosthesis, Pediatric, fNIRS, Brain activation

## Abstract

**Background:**

The purpose of the current study was to determine the influence of upper-limb prostheses on brain activity and gross dexterity in children with congenital unilateral upper-limb reduction deficiencies (ULD) compared to typically developing children (TD).

**Methods:**

Five children with ULD (3 boys, 2 girls, 8.76 ± 3.37 years of age) and five age- and sex-matched TD children (3 boys, 2 girls, 8.96 ± 3.23 years of age) performed a gross manual dexterity task (Box and Block Test) while measuring brain activity (functional near-infrared spectroscopy; fNIRS).

**Results:**

There were no significant differences (*p* = 0.948) in gross dexterity performance between the ULD group with prosthesis (7.23 ± 3.37 blocks per minute) and TD group with the prosthetic simulator (7.63 ± 5.61 blocks per minute). However, there was a significant (*p* = 0.001) difference in Laterality Index (LI) between the ULD group with prosthesis (LI = − 0.2888 ± 0.0205) and TD group with simulator (LI = 0.0504 ± 0.0296) showing in a significant ipsilateral control for the ULD group. Thus, the major finding of the present investigation was that children with ULD, unlike the control group, showed significant activation in the ipsilateral motor cortex on the non-preferred side using a prosthesis during a gross manual dexterity task.

**Conclusions:**

This ipsilateral response may be a compensation strategy in which the existing cortical representations of the non-affected (preferred) side are been used by the affected (non-preferred) side to operate the prosthesis. This study is the first to report altered lateralization in children with ULD while using a prosthesis.

*Trial registration* The clinical trial (ClinicalTrial.gov ID: NCT04110730 and unique protocol ID: IRB # 614-16-FB) was registered on October 1, 2019 (https://clinicaltrials.gov/ct2/show/NCT04110730) and posted on October 1, 2019. The study start date was January 10, 2020. The first participant was enrolled on January 14, 2020, and the trial is scheduled to be completed by August 23, 2023. The trial was updated January 18, 2020 and is currently recruiting

## Introduction

The Centers for Disease Control and Prevention (CDC) estimates that about 4 out of every 10,000 babies are born with upper-limb reductions every year in the U.S [1, 2]. In other parts of the world, such as Australia, Finland, and Canada reports indicate that 3.4 to 5.3 of 10,000 live-born children suffer upper-limb anomalies [3]. In the United States, however, there are many more unreported cases due to the lack of a mandatory reporting system of birth defects and child amputees. The use of upper-limb prostheses is the main treatment to restore function in children experiencing upper-limb reduction deficiencies (ULD) [1]. In addition, providing a functional prosthesis can be expensive, ranging in cost from $4000 to $10,000 for a body-powered prosthesis and $25,000 to $75,000 for an electronically driven prosthesis [4]. Due to the increased cost and lack of insurance coverage there is still many children who do not have access to a prosthesis [5–7]. Recent technological advances in computer-aided design (CAD) programs and additive manufacturing (i.e., 3D printing) [7], made it possible to design and manufacture child-friendly 3D printed prostheses that can be customized as the child growths with low-cost, lightweight, and desirable visual appearance [5–11].

### Pediatric population

The CDC indicated that children with upper-limb reduction deficiency (ULD) will face potential problems including, difficulties with normal development such as motor skills, needing assistance with daily activities such as self-care, limitations with certain movements, sports, or activities, as well as potential emotional and social issues because of physical appearance [1]. For children with ULD, the use of prostheses is directly related to the success of rehabilitation outcomes including development of motor skills, performance of activities of daily living and recreational activities, as well as improvements in self-esteem [1, 6, 7, 11]. However, increasing prosthetic use and reducing rejection and abandonment in the pediatric population remain challenging, with up to 58% rejection rate [12–14]. The reasons for pediatric rejecting rates include excessive weight, low visual appeal, low comfort, and lack of function [14–16]. While these factors are exclusive to the design of the prosthesis, previous literature suggest the involvement of specific neural control mechanisms that limit the functional use of these devices [12, 13, 15].

### Adult population

Although the reasons for rejecting a prosthesis in the adult population are similar to those for the pediatric population including the excessive weight, low comfort, and lack of function [17], the neural process and motor control parameters of rehabilitation outcomes in the pediatric population are significantly different [13, 15, 18]. However, there is a lack of data shown the neural mechanism of novel rehabilitation approaches in the pediatric population [12, 19]. Previous investigations in the adult population, however, have shown that the neural process underlying the acquisition of new motor skills using a prosthesis and the neuromuscular outcomes can have a profound impact in the development of alternative prosthetic rehabilitation approaches, such as the cross education of motor function [20, 21]. Cross education is the process of training the non-affected limb to enhance the motor performance of the affected, untrained limb [21]. Prosthetic simulators are devices used to mimic the function of a prosthesis often used to achieve cross education or within rehabilitative settings to assist with prosthesis familiarization when the affected limb is injured or in the early post-surgery stages [22–24]. Previous literature has also shown that a spectrum of tools from simple rods to prosthetic simulators can be embodied within the brain, which may affect the efficiency of an individual’s kinematics [25, 26]. These simulators can also be used in individuals with intact arms to examine in the cortical adaptations to novel tool use, such as the use of a prosthesis [26].

### Knowledge gap

There is a significant knowledge gap about the neural mechanism underlying the high rejection rate of upper limb prosthesis in children [12]. The foundational knowledge of neural plasticity of motor control in this population has been severely under studied [13, 15, 18]. The Neuronal Group Selection Theory (NGST) [18, 19, 27] states that the ensemble of cortical and subcortical systems is dynamically organized into neuronal networks. The structure and function of these neuronal networks are determined by development and behavior [18, 19, 27]. According to NGST, children with congenital unilateral upper-limb reductions may lack representation of the missing part of the limb in the cerebral cortex, leading to limited number of “motor repertoires” for the affected upper-limb [19, 27]. Therefore, early intervention in these children with limb reductions, such as prosthetic fitting and use, may lead to an enlargement of the primary neuronal networks located in the contralateral motor cortices of the affected limb. As a result, the early use of prosthetic limbs might lead to a larger repertoire of motor system and improve integration of the prosthesis into the sensory and motor system to facilitate prosthesis acceptance in children with limb reductions [12, 13, 15, 19, 27].

Previous investigations in adults have reported increases in strength, motor skills, motor learning, and motor performance in the affected, untrained upper limb after training the non-affected limb [20, 21]. However, the precise neural mechanism in children with ULD remains unclear [12, 19, 27]. Determining the specific role of each hemisphere in controlling the affected and unaffected limbs and how each hemisphere is involved in controlling and adapting to a prosthesis seems critically important. Thus, the purpose of the current study was to determine the influence of upper-limb prostheses and prosthetic simulators on brain activity and dexterity compared to typically developing control children. Based on the current literature [11, 20–22, 25, 28] it is hypothesized that: (i) Dexterity will not be significantly different between prosthesis and simulator groups and (ii) lateralization of the brain will be less pronounced for prosthesis users (due to less specialized neural organization).

## Methods

### Experimental design

An experimental group of children with unilateral upper-limb reduction deficiency on the left side. A sex- and age matched control group performed a gross manual dexterity task with the preferred (right) and non-preferred (left) sides while measuring motor cortical activity in both hemispheres. The experimental group performed the motor task wearing a prosthesis on the non-preferred side (affected left side) and similarly, the control group performed the same task wearing a prosthetic simulator on the non-preferred side (also left side). All children (experimental and control groups) showed right-hand preference.

### Subjects

Five children with congenital, ULD (3 boys, 2 girls, 8.76 ± 3.37 years of age) and five age- and sex-matched typically developing control subjects (TD; 3 boys, 2 girls, 8.96 ± 3.23 years of age) were enrolled. Two upper-limb deficient subjects had trans-radial reductions, and three had partial hand (trans-metacarpal) reductions (Table 1). All subjects ULD had left side impairment and showed right-hand preference. All typically developing children preferred their right hand when performing motor tasks. The preferred hand of both groups was determined by the repeated preference of their right hand during observed behaviors such as writing, drawing, throwing, and forward reaching tasks with and without the device as well as the results from the Handedness Questionnaire [29].

**Table 1:** Characteristics of research participants (n = 10)

ID	Gender	Age (years)	Preferred side	Reduction level	Affected side	Ability to pinch
Experimental group (congenital upper limb reduction deficiency)						
1	Girl	6.2	Right	Partial hand	Left	No
2	Girl	8.2	Right	Trans-radial	Left	No
3	Boy	11.1	Right	Partial hand	Left	No
4	Boy	5.1	Right	Trans-radial	Left	No
5	Boy	13.2	Right	Partial hand	Left	No
M ± SD		8.76 ± 3.37				
Control group (typically developing)						
1	Girl	6.4	Right	None	None	Yes
2	Girl	8.3	Right	None	None	Yes
3	Boy	11.3	Right	None	None	Yes
4	Boy	5.6	Right	None	None	Yes
5	Boy	13.2	Right	None	None	Yes
M ± SD		8.96 ± 3.23				

Experimental group presented congenital reduction deficiencies. Age was not significantly different

Inclusion criteria were children (male and female; aged 3–16 years) with congenital, unilateral upper-limb reductions of any digit, hand, arm, or shoulder. Any subjects with prior prosthesis experience were included only if they had not used a prosthesis for at least six months prior to conduction of the study. Exclusion criteria included upper extremity injury within past month, medical conditions that are contraindications for wearing a prosthesis (such as skin abrasions and musculoskeletal injuries of the upper limbs), as well as neurological or psychiatric disorders based on parent’s report. For the control subjects, all inclusion and exclusion criteria were identical aside from the presence of a congenital upper limb reduction.

All children were admitted to the study following informed assents or parental written consent as approved by the Institutional Review Board of the University of Nebraska Medical Center.

All subjects completed a medical history questionnaire. Parents and children were informed about the study and parents signed a parental permission form. For children aged 6–10 years, an assent was explained by the corresponding author and signed by the children and their parents. Additionally, detailed safety guidelines were given to parents of upper-limb deficient subjects regarding the use and care of the prosthesis.

### Gross manual dexterity task

The Box and Block Test was used to assess gross manual dexterity for the experimental and control group. The Box and Block Test has been suggested as a measure of unilateral gross dexterity [30, 31] and has been previously used to assess upper-limb prosthetic performance and motor learning [32]. Norms have been collected on adults with neuromuscular involvement and in typically developing children [30, 31]. The Box and Block Test consist in a wooden box dimensioned in 53.7 cm × 25.4 cm × 8.5 cm. The partition is placed at the middle of the box, dividing it in two containers of 25.4 cm each. There are 150 wooden cubes of 2.5 cm in size [30]. The Box and Block Test provides quantitative data regarding the gross dexterity of the affected and non-affected upper limbs [15, 16].

After providing instructions, the children were allowed a 15-s trial period prior to testing. Immediately before testing begins, the child was asked to place his/her hands on the sides of the box. When testing begins, each child is asked to grasp one block at a time, transport the block over the partition, and release it into the opposite compartment. This task was performed for 1 min in duration. A 30 s period of rest was given and the procedure was then repeated with the other hand. After testing, the blocks were counted. If a child transported two or more blocks at the same time, this was noted and subtracted from the total.

### Design and fitting of the prostheses and prosthetic simulators

#### Prostheses

**Fig. 1:**
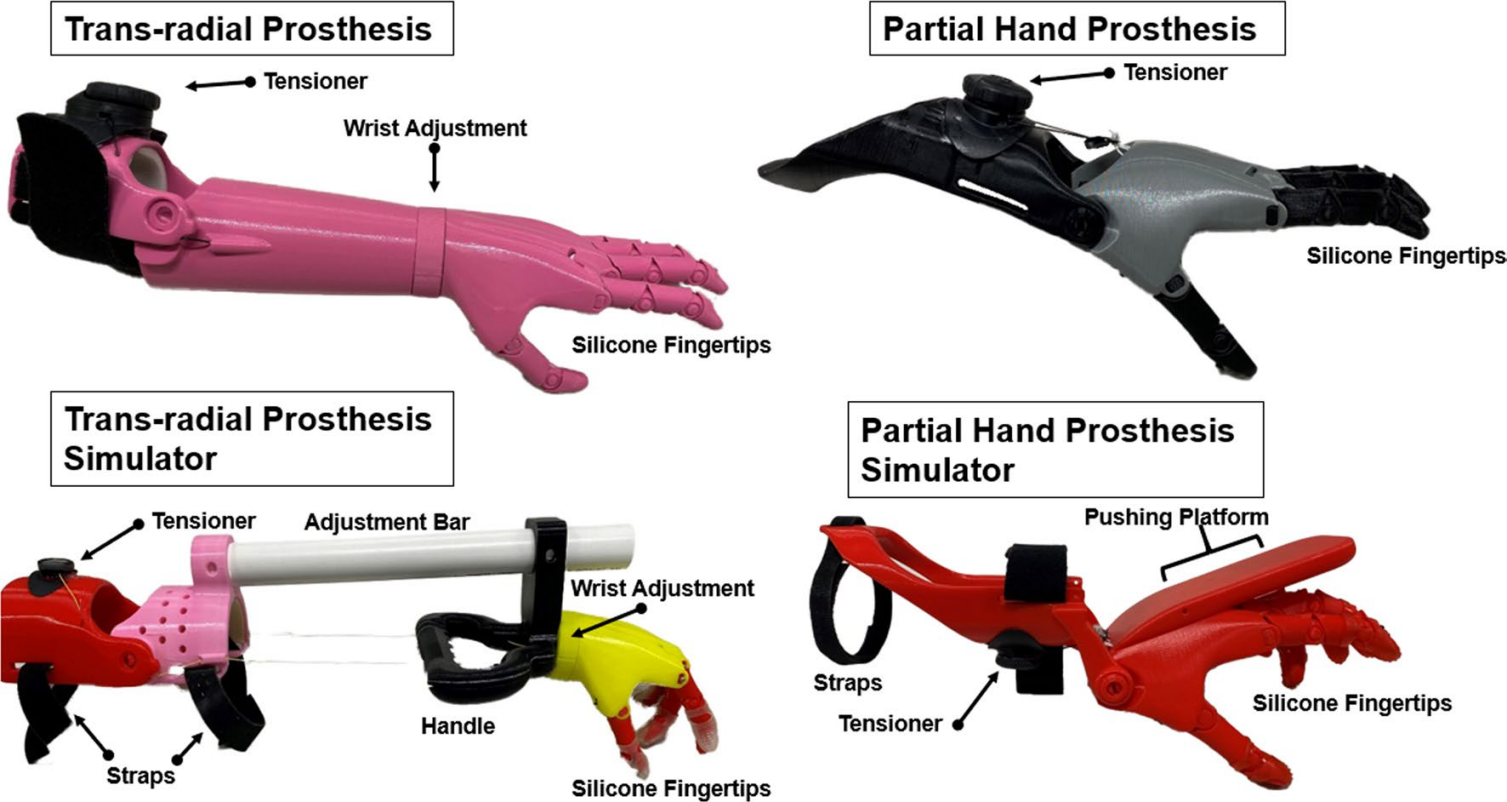
Description of prostheses and prosthetic simulators. The prosthetic simulators used in the study mimic the design and control mechanism of the prostheses. The partial hand prosthesis simulator allowed typically developing children to rest their existing hand on top of the simulator hand, with the wrist in slight extension. A pushing platform placed above the hand allowed wrist active flexion and passive extension to facilitate actuation of the hand. Similarly, the trans-radial simulators incorporated similar features than the trans-radial prosthesis with the addition of a handle that allowed typically developed children to actuate the device by elbow flexion

A modified version of the 3D-printed transitional partial hand and trans-radial arm prostheses named Cyborg Beast 2 was used in the study [6] (Fig. 1). The modified devices were designed using Autodesk Fusion 360 (Fusion 360, Autodesk, Inc., San Rafael, CA, USA) and were manufactured in Biomechanical Rehabilitation and Manufacturing Facilities located in the Biomechanics Research Building of the University of Nebraska at Omaha.

The partial hand and trans-radial prostheses used in the current investigation are classified as voluntary-closing devices [6] (Fig. 1). The voluntary closing terminal devices were custom scaled and fitted to a customized socket [5]. Specifically, the partial hand prostheses incorporated a simple hinge joint in the wrist for grip actuation driven by wrist flexion, while trans-radial design included a similar hinge mechanism at the elbow and was driven by elbow flexion. To increase visual appeal all hands have been designed to incorporate five fingers, each with 2 degrees of freedom. The index finger and thumb are oriented in opposition to facilitate cylindrical grasp and tip pinch. Silicone fingertips were added to provide enhanced traction and pliability for grasping activities. Elastic cords placed inside the dorsal aspect of the fingers provided passive finger extension. Finger flexion was produced by nylon cords embedded in the palmar surface of each finger and actuated by flexion of the wrist (partial hand prosthesis) or elbow (trans-radial prosthesis). The finger and thumb were oriented in opposition to facilitate cylindrical grasp and pinch. A BOA dial tensioner system (Mid power reel M3, BOA Technology Inc., Denver, Colorado) allowed regulating the tension of the cables controlling the finger flexion (Fig. 1).

#### Prosthetic simulators

The prosthetic simulators used in the study used identical design and actuation methods to the prosthetic devices (Fig. 1). The primary function of these prosthetic simulators was to replicate the actuation and general function of the partial hand and trans-radial prostheses in typically developing children, thus the prosthetic simulator was placed in the non-dominant arm of the TD group. The partial hand simulator allowed typically developing children to rest their existing hand on top of the simulator hand, with the wrist in slight extension. A pushing platform placed above the partial hand simulator, was strapped to the hand to immobilize the fingers and allow for wrist active flexion and passive extension to facilitate actuation of the hand. Similarly, the trans-radial simulator incorporated similar features than the prosthetic device with the addition of a handle that allowed typically developed children to actuate the device by elbow flexion (Fig. 1).

### 3D printing specifications

Desktop 3D printers (Ultimaker 2 Extended+, Ultimaker B.V., Geldermalsen, the Netherlands) were used for the manufacturing of the devices. The prosthesis was manufactured using PLACTIVE™ (PLACTIVE™ 1% Antibacterial copper additive, Copper3D Inc, Santiago, Chile) which is a high quality polylactic acid polymer PLACTIVE™ physical and mechanical properties are optimal for prosthetic applications [33].

All parts were printed at 40% infill (hexagon pattern), 50 mm/s print speed, 150–200 mm/s travel speed, 50 °C heated bed, printing temperature of 200 °C, 0.15 mm layer height, and 1 mm shell thickness. Post-processing consisted in support removal and filing of rough areas in the joints and prosthetic socket area in contact with the skin.

### Functional near-infrared spectroscopy

**Fig. 2.**
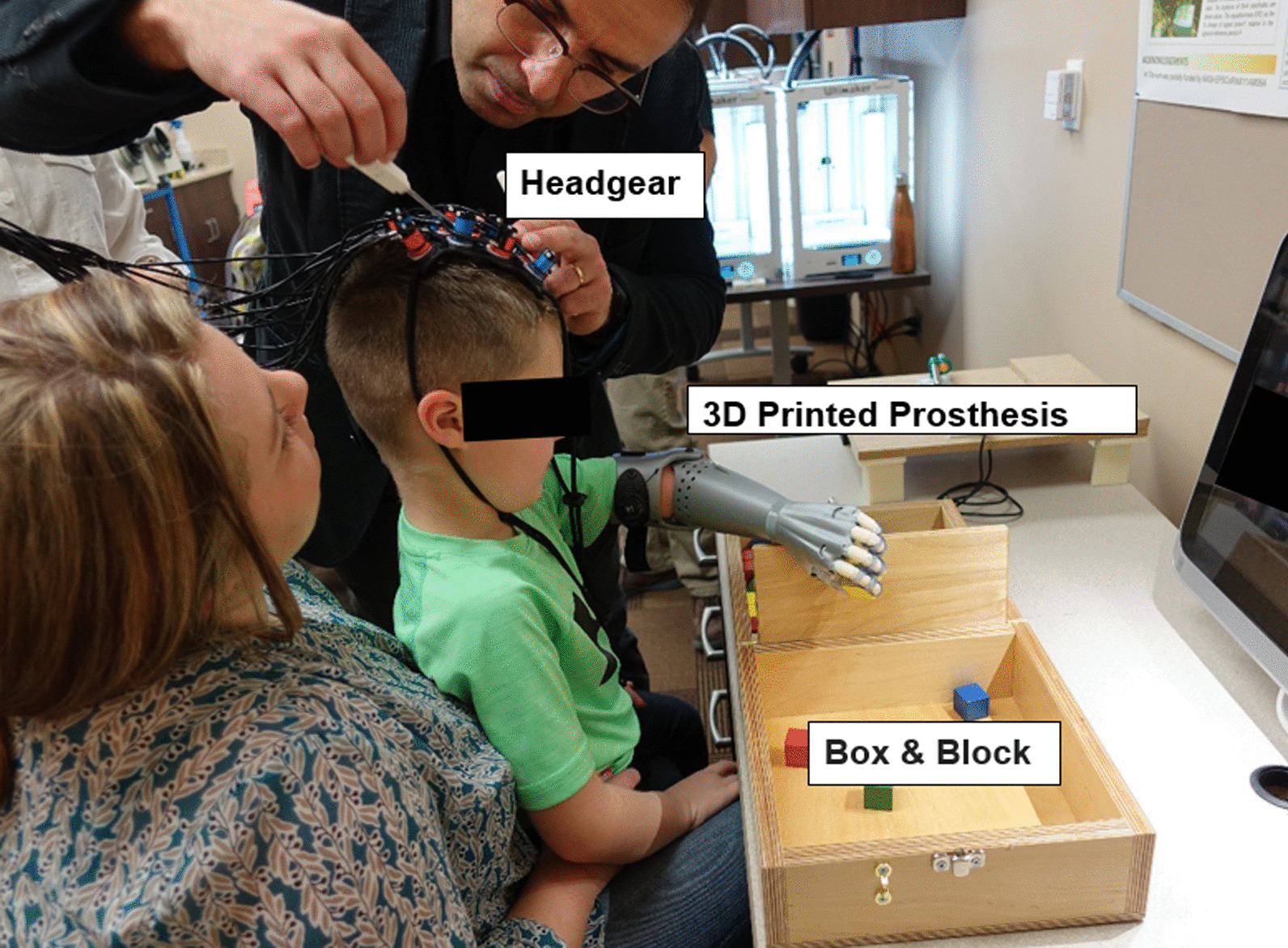
Placement of the functional near-infrared spectroscopy (fNIRS) head set and probe adjustment

**Fig. 3:**
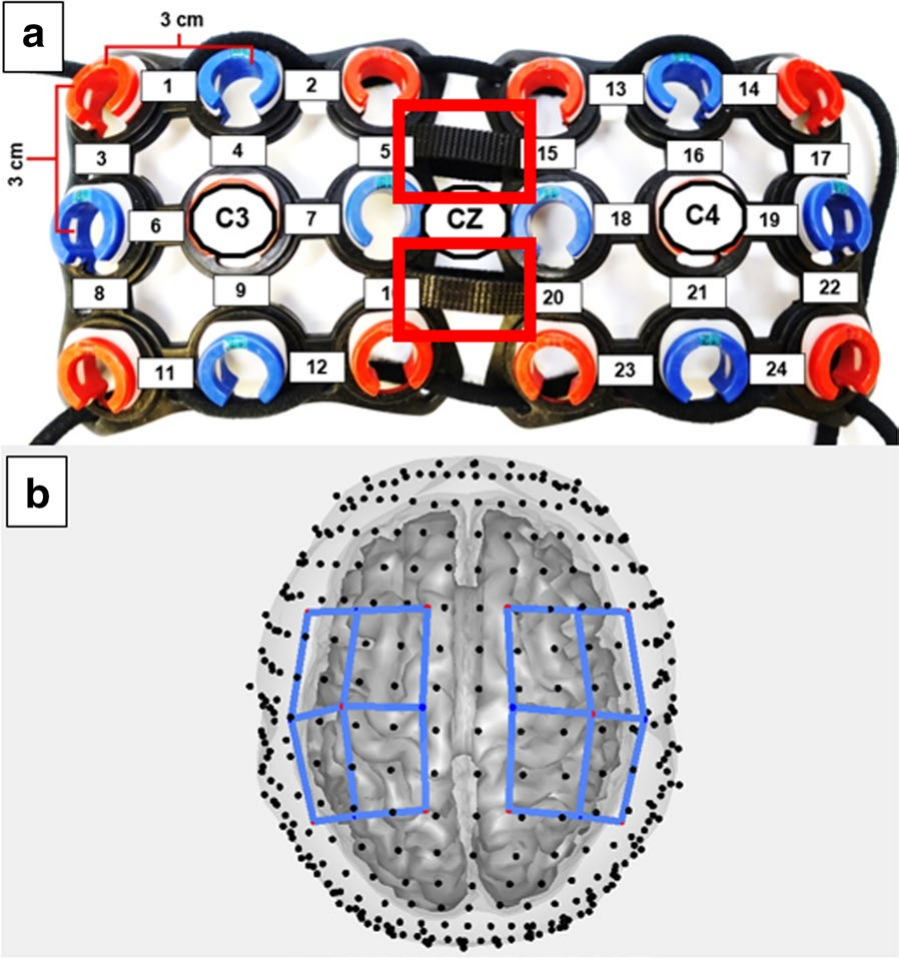
**a** Adjustable headgear and channels arrangement. The headset was centered at the vertex (Cz) and lateral channels placed over the C3 and C4 motor cortex landmarks associated with motor activity of the hand and arm movements. Red rectangles show the adjustable Velcro straps used to accommodate different head sizes. **b** Visualization of headgear after virtual registration to subject brain model. Blue lines indicate placement of the headgear over the brain

A continuous wave 24-channel fNIRS system (Hitachi ETG-4000, Hitachi Medical Corporation, Tokyo, Japan) was used to non-invasively investigate the changes in oxygenated (HbO) and deoxygenated (HbR) hemoglobin concentrations in the sensorimotor cortex and nearby brain areas during a gross manual dexterity task (Fig. 2). The adjustable headgear (Fig. 3) was positioned on the head following the 10–20 international system [34] so that the center of the headgear was aligned with the vertex (Cz) and lateral channels cover the area around the C3 and C4 landmarks (including precentral and postcentral gyri; Fig. 3), which have been shown to detect motor activity and sensory information related to hand and arm movements [35, 36]. The probe set used consisted of 10 sources and 8 detectors separated by 3 cm in a cap, which housed mounting geometries for the optodes (Fig. 3). Adjustable straps present in the center of the cap allowed for appropriate probe positioning (Fig. 3). The probe holders and their position on the head ensured stable optical contact with the subjects’ scalp for all optodes. For the left side, the 3 × 3 source-detector set was centered on C3 and the right side 3 × 3 source-detector set was centered on C4.

The measurement principles are based on the modified Beer–Lambert law with a differential path length correction of 6 and partial volume correction of 1/60 for both wavelengths [37]. The ETG-4000 utilizes two kinds of near-infrared light (695 and 830 nm). The sampling rate for the recording was 10 Hz and recordings were transferred from the ETG-4000 to an encrypted hard drive.

### Data analysis

#### Probe registration

To register a fNIRS probe a set of landmarks are defined relative to the fNIRS sensors in the two-dimensional space based on 10–20 coordinates. Using this information, the probe is then registered to the Colin27 atlas (stereotaxic average of 27 T1-weighted MRI scans) [38], which generates a layered head model (skin, skull, cerebral spinal fluid, gray/white matter). The registration of the fNIRS probe to the brain model is done by rescaling the brain to match the estimated head circumference of the subject.

#### Region of interest analysis

Regions-of-interest, which look at localized brain activation, are defined using anatomical registration. For three-dimensionally registered probes, the expected relative sensitivity of each fNIRS source-to-detector channel to anatomical parcellation labels can be used to define a weighted region-of-interest based on the optical forward model. This optical forward model defines the sensitivity of the measurements in channel space to underlying changes in the brain space. For anatomical regions-of-interest, the optical forward model and a brain-space region mask define the contrast vector in channel-space. For statistical testing of the region-of-interest, this contrast vector defines the expected response in channel space given the region in brain space. Thus, it is possible to test the null hypothesis that the signal from the region-of-interest is equal to zero. The result is a series of weighted source-detector pair activations which are correlated to anatomical brain regions for each subject, which can then be compared to assess common patterns of activation and involved brain regions which are statistically significant [39–41].

#### Cortical activation

**Fig. 4:**
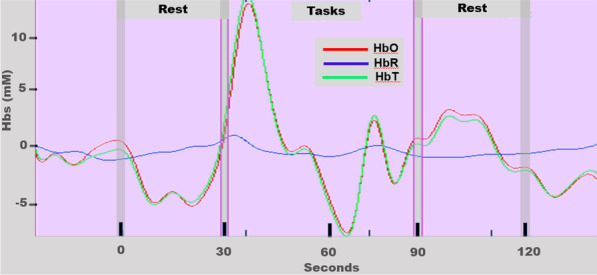
Functional near-infrared spectroscopy (fNIRS) filtered waveform from the motor cortex of the left hemisphere of the experimental group (Subject 2 in Table 1)

**Fig. 5:**
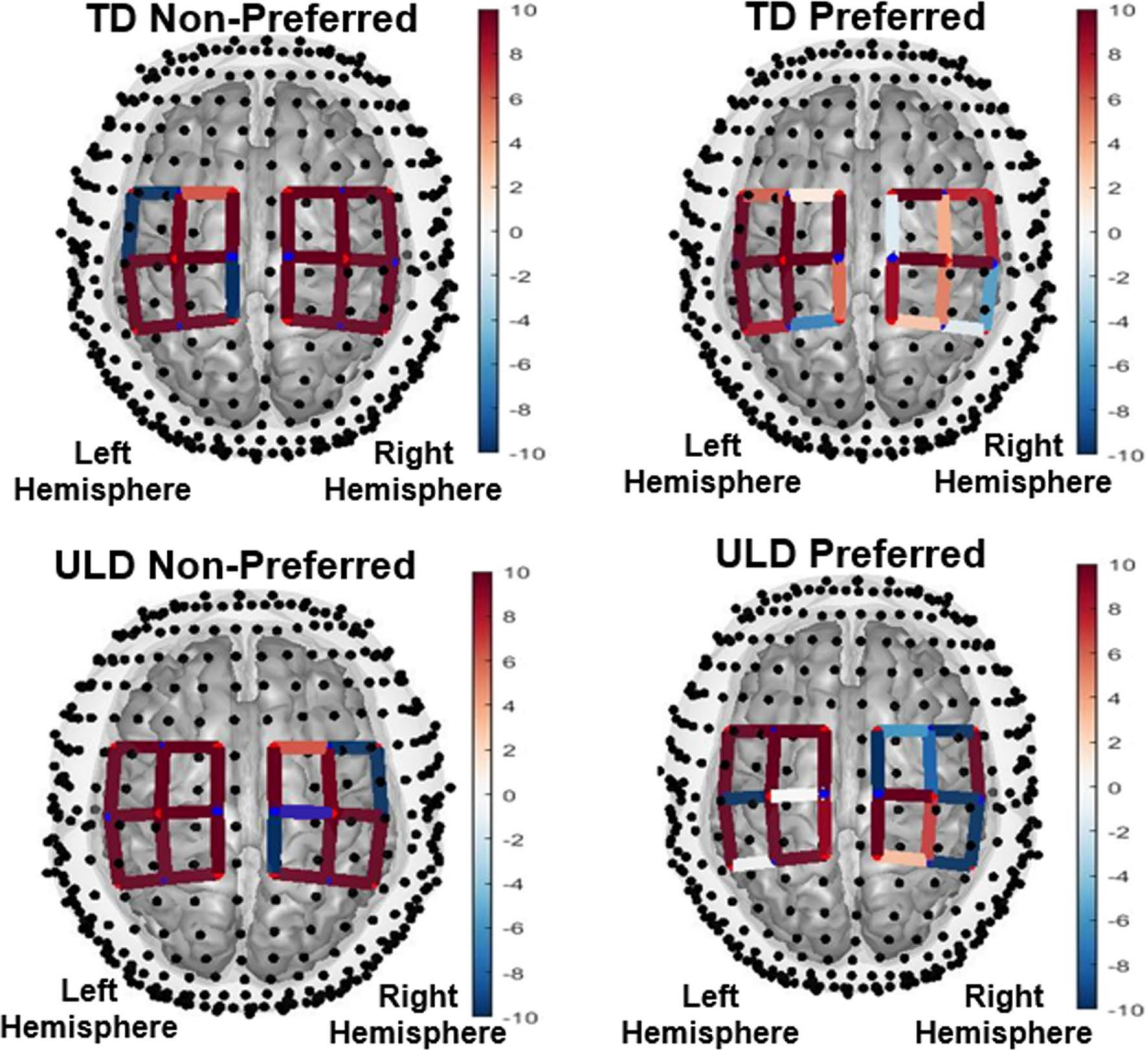
Visualization of brain activity patterns from a typically developing (TD) child (Control group ID: 5) and a child with unilateral limb deficiency (ULD; Experimental group ID: 5). The left hemisphere from the children with ULD showed a significant ipsilateral activation

The NIRS Brain AnalyzIR Toolbox [40] was used to analyze the fNIRS data. The AnalyzIR Toolbox is a Matlab (The MathWorks, Inc., Natick, Massachusetts, United States) based statistical and visualization package which is able to analyze time-series fNIRS data through the use of general linear models (GLMs) such as linear regression [39–41]. The assumption of GLM is that the brain’s response to a task condition is linearly additive and consistent across trials [39, 42]. The GLM model is described by the equation $\Delta [Hbx]=X\cdot \beta + \varepsilon$, where $\Delta [Hbx]$ represents the measurement vector, therefore the changes in concentration of HbO and HbR (Fig. 4) in a given brain region, while *X* stores information regarding event onset and termination including the design matrix encoding the timing of stimulus events, $\beta$ defines the unknowns in the model representing the weighted regression coefficients for a particular source-detector channel. and $\varepsilon$ represents measurement error [39]. The design matrix was constructed from the convolution of the stimulus timing and duration with a canonical hemodynamic response function (see details in Barker et al. [43]). The purpose of this linear regression is to estimate these weighted regression coefficients based on the data vector Y ($\text{i.e}., \Delta [Hbx]$) and the design matrix X. If the weight $\beta$ associated with a particular regressor (e.g., a specific task) is statistically nonzero, then that regressor is important in modeling the data. Thus, the statistical map of these weights associated with task components is generally interpreted as indicating the brain regions that statistically change based on the task (e.g., brain activity). Thus, in this analysis, there is no preprocessing needed. Instead, the two major sources of confounding noise, physiological noise and motion artifacts, were dealt with statistically within the GLM [39]. To reduce systemic physiology and motion-induced artifacts, an iteratively auto-regressively whitened, weighted least-squares model was used to solve the general linear equation. This regression model uses an nth order auto-regressive filter determined by an Akaike model-order selection to whiten both sides of the GLM expression [44]. Specifically, for the subject level analysis the regression coefficients (β) and their error-covariance (Covβ) were estimated, and used to define statistical tests between task conditions or baseline. To investigate if performing the motor task elicited a significant brain activation compared with the resting period we used a GLM with a boxcar function of the timing of the motor task as a regressor [43]. The regression model was solved sequentially for each data file for each research participant. All source-detector pairs within a file were solved concurrently providing a full covariance model of the noise, which was used in the group-level analysis. T-tests were used to determine if the regression coefficients were statistically non-zero (Figs. 4 and 5).

For each condition (ULD and TD preferred and non-preferred side with device), group-level analysis was performed using a linear mixed effects model, using the task-related regression weights (β) from the first-level GLM as the dependent variable and subject as a random effect. A modified version of the MATLAB function fit LME (linear mixed effects model estimator) was used to solve the weighted maximum likelihood estimate of the parameters. The model was whitened using the error covariance (Covβ) of the first level GLM model. To control for multiple comparisons, a Benjamini–Hochberg [45] false discovery rate (FDR) correction was used with the significance level set at 0.05 (p ≤ 0.05) [46]. In summary, the AnalyzIR Toolbox [40] was able to compute statistical significance (*T-*test) in numerous models for both subject- and group-level statistics on a voxel by voxel basis, allowing for visualization of cortical hemodynamic responses with very good spatial resolution and correlation to specific brain regions [39, 40, 42, 43]. Through the automatic weighting of regression coefficients for each source-detector pair, a partial intensity of each pair’s signal can be inferred as being caused by brain activity in a specific region with good accuracy to the registered and scaled Colin27 brain atlas model using a region of interest (ROI) analysis [40]. This analysis allows for the evaluation of less “coarse” brain regions than more traditional analyses, and acts as a supplement to the mean value assessments for HbO and HbR (Fig. 5).

#### Laterality Index

The Laterality Index (LI) was used to reveal hemispheric dominance using the following formula:

$${\text{LI}}=\frac{Hb{O}_{l}-Hb{O}_{r}}{Hb{O}_{l} +Hb{O}_{r}}$$

**Fig. 6:**
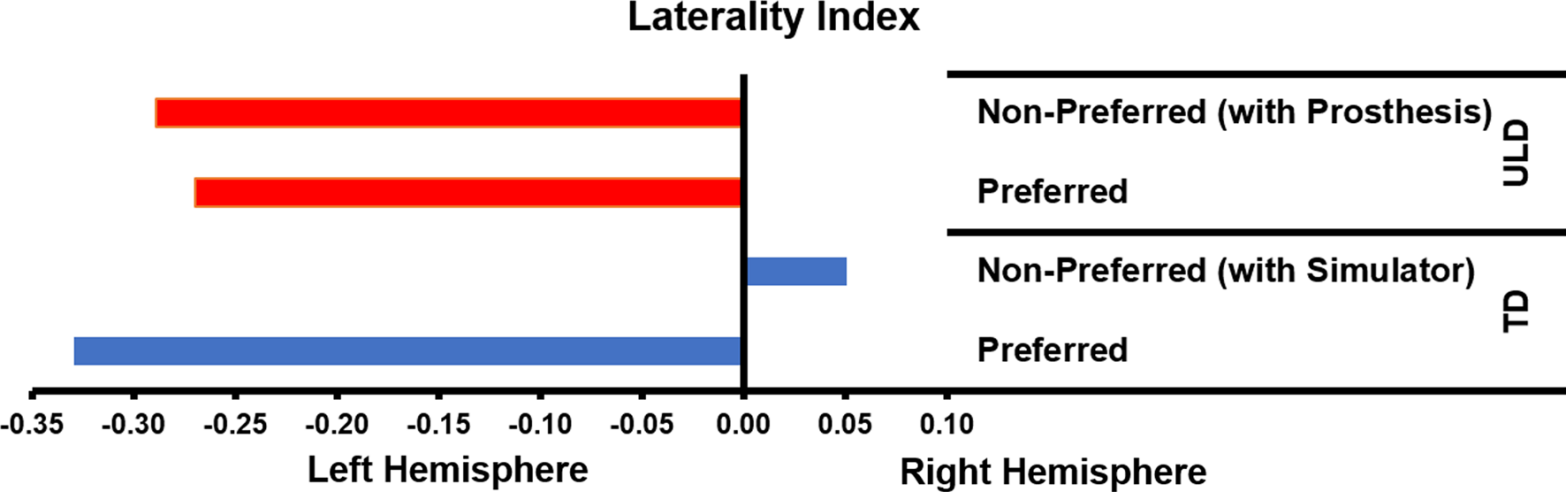
Laterality indices of children with upper-limb deficiency (ULD) and typically developing (TD) children during the performance of a functional task with preferred (right) and non-preferred hands (left for TD and affected for ULD)

In this equation, HbO_l_ represents a left hemisphere channel and HbO_r_ indicates the channel from the right hemisphere in the corresponding pair. The LI normalizes cortical activation differences between channels, thereby revealing which hemisphere experienced a larger change during the task. Negative values indicate right-hemisphere dominant activations, while positive values indicate a left hemisphere dominant activation. Thus, an LI value of “− 1” represents complete left hemisphere dominant activation, an LI value of “+ 1” corresponds to complete right-hemisphere dominant activation and an LI of “0” reflects bilateral activation (Fig. 6) [47].

#### Statistical analysis

A Shapiro–Wilk test was performed to analyze all data for normality with a C.I of 95%, with the null hypothesis of this test stating that the LI using the limb of interest (non-preferred with device) is normally distributed within the sample population. Similarly a Levene’s Test of Homogeneity was also conducted to assess variance of the LI using the non-preferred limb between groups.

A two-way repeated measures ANOVA [2 × 2; hand (non-preferred side with device versus preferred side) × group (ULD versus TD)] was used to test hand × group interactions. The TD group used a prosthetic simulator equivalent to the prosthesis use by the ULD group in the same side LI values used as the dependent variable. An alpha value of 0.05 was considered statistically significant for all comparisons.

To determine the effectiveness in rehabilitation on functional outcomes we calculated the Minimal Clinically Important Difference (MCID) [48, 49]. To estimate the MCID, which is the smallest amount of change in an outcome that might be considered clinically important we multiplied the pooled SD’s of the two group mean for preferred and non-preferred hands for manual gross dexterity and laterality index by 0.2 [48, 49].

## Results

### Group characteristics

Demographic characteristics of the sample are outlined by group status in Table 1. The groups did not differ in age, or gender. The prosthetic simulators used in the typically developing children group were matched to the hemisphere and level of amputation of the age- and sex-matched to ULD subjects. All ULD subjects did not used a prosthesis for a minimum of 6 months before participation in the study. All subjects were right-handed.

The Shapiro–Wilk indicated that were non-significance results for the ULD (p = 0.639) and TD (p = 0.755) groups demonstrating normally distributed data. The Levene’s Test of Homogeneity using a 95% confidence interval indicated that for all the trials tested there were no significant differences (p = 0.197) from the null hypothesis. These results suggest that all conditions were normally distributed and that the variances were homogenous between subject groups within the experiment.

### Gross manual dexterity performance

There was a significant hand × group interaction for gross manual dexterity performance, F(1,4) = 96.034, p = 0.002. Post-hoc analyses revealed that for the preferred hand, the ULD group performed significantly lower (37.40 ± 6.65 blocks per minute) than the TD group (47.50 ± 6.2 blocks per minute). In agreement with our hypothesis, there was no significant difference (*p* = 0.948) in gross manual dexterity between the ULD group with prosthesis (7.23 ± 3.37 blocks per minute) and TD group with simulator (7.63 ± 5.61 blocks per minute).

The MCID in manual gross dexterity between groups for preferred and non-preferred hands was 1 block per minute.

### Lateralization of brain activity

Significant differences from baseline (p < 0.05) were found for the superior aspects (left and right) of the precentral gyrus (M1) for ULD and TD groups (Fig. 5 and 6).

There was a significant hand x group interaction for LI, F(1,4) = 51.450, p = 0.002. Post-hoc analyses (Tukey’s HSD) performed to decompose the model revealed that for the preferred hand, there were no significant differences (*p* = 0.2) between the ULD group (LI = − 0.2697 ± 0.0550) and TD group (LI = − 0.3293 ± 0.0879) LI values. For the non-preferred hand, however, there were significant differences (*p* = 0.001) between the ULD group with prosthesis (LI = − 0.2888 ± 0.0205) and TD group with simulator (LI = 0.0504 ± 0.0296) LI values, which was in agreement with our hypothesis showing a less pronounce lateralization for prosthetic users (Table 3). Furthermore, a significant difference (*p* = 0.001) was found between the preferred (LI = − 0.3293 ± 0.0879) and non-preferred hand with simulator (LI = 0.0504 ± 0.0296) for the TD group. No significant difference (*p* = 0.4) was found between the preferred (LI = − 0.2697 ± 0.0550) and non-preferred with prosthesis (LI = − 0.2888 ± 0.0205) for the ULD group.

As shown in Fig. 3, the TD group presented preferential activation in the contralateral motor cortex while performing the motor task. In contrast, the ULD group showed preferential activation in the ipsilateral motor cortex when using the non-preferred side (affected side with prosthesis). Thus, the ULD group was found to have significant ipsilateral dominance for the non-preferred hand with the prosthesis when compared to the TD group using the simulator (Figs. 5 and 6).

The MCID in LI between groups for the hemispheres controlling the preferred and non-preferred hands were 0.0146 and 0.0051, respectively.

## Discussion

The major findings of the present investigation are in agreement with our hypotheses indicating a non-significant difference in gross manual dexterity between prosthetic and prosthetic simulator groups, as well as a less pronounced brain lateralization in children with ULD, resulting in a significant ipsilateral control when using a prosthesis.

Children with ULD, unlike the control group, showed significant activation in the ipsilateral motor cortex while performing a gross manual dexterity task using a prosthesis (Fig. 3). To control for device use, the current investigation included a control group of typically developing children using a prosthetic simulator (Fig. 1) that was biomechanically equivalent to the prosthesis used by the experimental group (Fig. 1). The gross manual dexterity task performance was the same for both groups (Table 2), suggesting that the degree of skills using the prostheses or simulator was also similar. This type of ipsilateral contribution has been reported in children with total hemispherectomy [50], adults with internal capsular stroke [50] and adults with unilateral acquired amputations [51–53], but not yet reported in children with ULD.

**Table 2:** Box and Block Task performance

	Task performance (blocks moved per minute)	
	ULD	TD
Preferred hand	37.40 ± 6.65*	47.50 ± 6.20
Non-preferred hand with device	7.23 ± 3.37	7.63 ± 5.61
SD_Pooled_	6.43 (preferred) 4.63 (non-preferred)	
MCID	1 (preferred) 1 (non-preferred)	

ULD used a prosthesis in the non-preferred hand with a congenital reduction. TD use a prosthetic simulator in the non-preferred hand to match ULD. TD only used the simulator in the non-preferred hand, their preferred hand performed without a simulator

*MCID* Minimal Clinically Important Difference

*Significant differences (p = 0.020)

This ipsilateral response is consistent with recent findings in adults with acquired amputation of the preferred right hand [51]. Philip et al. [51], showed that the ipsilateral (left hemisphere) motor cortex plays a functional role in non-preferred hand motor learning and motor performance, specifically through experience-dependent changes in limb trajectory control (i.e., smoothness) [51, 54]. Similarly, a previous investigation by Hamzei et al. [52], presented structural and functional brain imaging data for seven cases (6 acquired and 1 congenital) of adults and children with acquired amputations and an adult with congenital reduction deficiency. Specifically, for a 22 years old subject with right congenital ULD showed ipsilateral sensorimotor cortex activation during right stump movements [52]. Reilly and Sirigu [53] found that one of four adults with upper limb reduction deficiency reported sensations in the affected limb evoked during transcranial magnetic stimulation of the ipsilateral hemisphere. Thus, there is some evidence suggesting that ipsilateral control may play a role in motor control strategy used by individuals with acquired or congenital ULD and for patients with congenital ULD it seems to be mostly influenced by previous experiences [51] that presumably occurred during critical development stages [28, 55].

Based on previous literature [13, 28, 51–56], the significant contribution of ipsilateral motor areas (Fig. 3) in the children with ULD found in the present investigation may suggest a functional role of the left hemisphere to improve performance as a compensatory strategy during critical development stages that may be influenced by prosthetic use [28]. The potential mechanism of action for the ipsilateral motor control may be related to the previously reported reduced levels of the inhibitory amino acid neurotransmitter gamma amino butyric acid (GABA) found in the motor cortex of individual with congenital ULD [56, 57], and reported to occur bilaterally after ischemic nerve blocking [20]. This decreased inhibition may enable or “unmask” [56] normally silenced, less specific inputs in the ipsilateral hemisphere, such as those originating from the affected limb using the prosthesis [28]. This decreased inhibition is greater at early developmental periods during childhood (i.e., critical periods) [55] in which the brain is more plastic and may also explain how individuals with congenital ULD can populate the neglected brain territories with other brain representations of cortically distant inputs of functional body parts [56] or artificial limbs to increase overall function [28]. This compensatory strategy is supported by investigations reporting improvement in performance of adults with amputations driven by limb trajectory smoothness controlled by the ipsilateral dominant hemisphere [51, 54] and a study showing that prolong prosthetic use in activities of daily living facilitate the recruitment of areas of the motor cortex normally devoted to the missing hand of adults with congenital ULD [28].

The NGST proposes that motor development is characterized by two phases of variation: primary and secondary [13, 15, 18]. During the primary variability phase that is present before the child is one year of age, motor activity is variable and not based on environmental conditions. In the secondary variability phase also called “experiential phase”, present after one year of age until adolescence, the child learns to select on the basis of active practice from a “variable movement repertoire” the most efficient motor strategy in each specific situation. However, it takes until adolescence before secondary variability of all motor functions obtains its adult configuration [13, 15, 18]. According to this theory children with ULD may lack representation of the missing part of the limb in the cerebral cortex [13, 15, 18]. Thus, it has been speculated that the child may have a limited number of “motor repertoires” for the affected upper-limb [13]. It has been suggested that intervention in these children at an early age, such as prosthetic fitting and use, may lead to an enlargement of the primary neuronal networks located in the cortical area involved with motor control of the affected limb [13, 15, 18] possibly facilitating the representation of the artificial limb on areas of the motor cortex normally devoted to the missing limb [28]. Under this framework, it seems conceivable that the significant contribution of ipsilateral motor pathways (Figs. 5, 6) in children with ULD found in the present investigation may be a compensation strategy in which the existing cortical representations of the non-affected (preferred) side are been used by the affected (non-preferred) side to operate the prosthesis [20, 21]. This rational is consistent with mirror movements [58] observed in three of the children in the present investigation (Subjects 1, 2 and 4). It has been reported [58] that mirror movements may originate ipsilaterally by uncrossed fast-conducting corticospinal tracts that descends during voluntary movements from the hand motor cortex area to the ipsilateral side of the spinal cord. Thus, it can be speculated that this ipsilateral projection could depend on either a branching of crossed cortico-spinal fibers or a separate ipsilateral cortico-spinal projection [58].

It can be hypothesized that this initial ipsilateral compensation during critical periods can be modified by prosthetic use. Thus, if the child uses the prosthesis extensively, it would facilitate the representation of the artificial limb on areas of the motor cortex normally devoted to the missing limb [28]. Recent findings [28] suggest that if a prosthesis is used frequently it can be neurophysiologically “embodied” supporting the notion that early prosthetic intervention in children with ULD, may facilitate an enlargement of the primary neuronal networks located in the cortical area involved with motor control of the affected limb. In theory, this may lead to a larger repertoire of motor strategies and integration of the prosthesis into the sensory and motor control of the child, facilitating prosthesis acceptance and embodiment [13, 15].

The potential rehabilitation applications of the results found in the present investigation are aligned with the bilateral and cross activation hypotheses from the cross-education theory [21]. Cross education is the process of training the non-affected limb to enhance the motor performance of the affected, untrained limb. Several investigations have reported increases in motor skills, motor learning, and motor performance in the affected, untrained upper limb after training the non-affected limb, but the precise neural mechanism in children has not been clarified [21]. The ipsilateral dominance found in the present investigation may provide an opportunity to effectively train the non-affected side to improve the functional performance of the affected side [20, 21]. These findings are not conclusive, however within the limitations of the present study, our results may suggest the potential use of prosthetic simulators to assist non-prosthetic users familiarizing with the device and potentially lowering the rejection and abandonment rate [12–14, 17]. These findings may provide justification for the use of rehabilitation paradigms that includes cross education elements during critical periods in children with unilateral congenital limb loss. By potentially stimulating the hemispheric region of the missing limb using a prosthetic simulator, it can be inferred that children with ULD may be less likely to reject and abandon their final prosthesis, however more research in this area is needed.

The main limitations of the present study are related to the small number of children with ULD participating in the study (n = 5), age difference (5 to 13 years of age), and the different reduction level, including partial hand (n = 3) and trans-radial (n = 2) reductions. The small sample size, wide age range difference, and different reduction levels may have introduced inter-subject variability in the neural and motor parameters assessed in the current study. To partially control for these limitations, we included an age and sex-matched control group using a prosthetic simulator equivalent to the prosthesis used by the children with ULD. In addition, we only included children with congenital unilateral left upper limb deficiency, thus right-hand dominant and a control group also right-hand dominant. These specific requirements resulted in a low sample size for the experimental group (n = 5). Despite all these limitations, our statistical significant results for our neurological parameter, LI (Table 3) were well above the smallest amount of change in an outcome that might be considered clinically important or MCID [48, 49].

**Table 3:** Laterality Index individual and mean values

	ULD		TD	
Subject ID	Preferred	Non-preferred with prosthesis	Preferred	Non-preferred with simulator
1	− 0.2270	− 0.3104	− 0.4104	0.0868
2	− 0.2490	− 0.2583	− 0.4233	0.0310
3	− 0.3658	− 0.2997	− 0.3284	0.0109
4	− 0.2480	− 0.2969	− 0.2550	0.0618
5	− 0.2587	− 0.2788	− 0.2293	0.0616
M ± SD	− 0.2697 ± − 0.0550	− 0.2888 ± 0.0205^a^	− 0.3293 ± 0.0879^a^	0.0504 ± 0.0296
SD_Pooled_	± 0.0733	± 0.0254		
MCID	0.0146	0.0051		

^a^Significant mean differences

*MCID* Minimal Clinically Important Difference

Future investigations should examine the longitudinal effect of prosthesis use and the effectiveness of using prosthetic simulators in neural and motor control parameters of children with ULD and acquire limb loss. Furthermore, other brain imaging methods, such as structural and functional magnetic resonance imaging and proton magnetic resonance spectroscopy should also be used to examine how prosthesis use influence different brain structures and function, as well as the concentration of GABA in the brain of children with congenital and acquire limb loss. Further analysis may help explain the mechanism describing how children with congenital or acquire limb loss can populate the neglected brain territories with the representation of other inputs, such as artificial limbs.

## Conclusion

In conclusion, the present investigation showed that children with congenital upper-limb reduction deficiency, unlike the control group, showed significant activation in the ipsilateral motor cortex on the non-preferred side using a prosthesis during a gross manual dexterity task. This strong contribution of ipsilateral motor pathways may suggest a functional role of the left hemisphere to improve performance as a compensatory strategy during critical development stages. It can be speculated that the potential mechanism of action for the ipsilateral motor control may be related to the reduced bilateral level of GABA found in the motor cortex of individual with congenital ULD. This decreased inhibition may enable or “unmask” normally silenced, less specific inputs in the ipsilateral hemisphere, such as those originating from the ipsilateral affected limb using the prosthesis. This ipsilateral response may be a compensation strategy in which the existing cortical representations of the non-affected (preferred) side are been used by the affected (non-preferred) side to operate the prosthesis.

## Data Availability

Individual values are reported in Tables 1 and 3. The code to analyzed the fNIRS signal is available from the corresponding author on reasonable request.

## Abbreviations

ULD: Upper-limb reduction deficiencies
TD: Typically developing
CDC: Centers for Disease Control and Prevention
fNIRS: Functional near-infrared spectroscopy
3D: Three dimensional
LI: Laterality Index
CAD: Computer-aided design
NGST: Neuronal Group Selection Theory
HbO: Oxygenated hemoglobin
HbR: Deoxygenated hemoglobin
GLM: General linear model
β: Regression coefficients and their error-covariance
Covβ: Regression coefficients error-covariance
ANOVAs: Analysis of variance
GABA: Gamma amino butyric acid
MCID: Minimal Clinically Important Difference
